# Splenic irradiation-induced gastric variceal bleeding in a primary splenic diffuse large B-cell lymphoma patient: a rare complication successfully treated by splenectomy with short gastric vein ligation

**DOI:** 10.1186/1477-7819-10-150

**Published:** 2012-07-16

**Authors:** Ying-Chu Lin, Hung-Chieh Chen, Shao-Bing Cheng, Wen-Li Hwang, Ren-Ching Wang, Chieh-Lin Jerry Teng

**Affiliations:** 1 Department of Medicine, Division of Hematology/Oncology, Taichung Veterans General Hospital, 160, Section 3, Chungkang Road, Taichung, 407, Taiwan; 2 Department of Radiology, Taichung Veterans General Hospital, 160, Section 3, Chungkang Road, Taichung, 407, Taiwan; 3 Department of Surgery, Division of General Surgery, Taichung Veterans General Hospital, 160, Section 3, Chungkang Road, Taichung, 407, Taiwan; 4 Department of Pathology, Taichung Veterans General Hospital, 160, Section 3, Chungkang Road, Taichung, 407, Taiwan; 5 Department of Life Science, Tunghai University, 181, Section 3, Chungkang Road, Taichung, 407, Taiwan; 6 Department of Medicine, Chung Shan Medical University, 110, Section 1, Jianguo North Road, Taichung, 402, Taiwan

**Keywords:** Irradiation, Lymphoma, Splenectomy, Thrombosis

## Abstract

Primary splenic diffuse large B-cell lymphoma (DLBCL) is a rare clinical condition, which is generally treated by six to eight cycles of chemotherapy involving a combination of rituximab and the cyclophosphamide, adriamycin, vincristine, and prednisolone (CHOP) regimen. However, the treatment for chemorefractory primary splenic DLBCL remains controversial. Therapeutic splenic irradiation (SI) might be a reasonable and possibly the only treatment option with curative intention for patients with chemorefractory primary splenic DLBCL. However, the efficacy and safety of therapeutic SI are unclear. Herein, we present the case of a primary splenic DLBCL patient who was refractory to multiple chemotherapy regimens but achieved complete remission after administration of therapeutic SI. However, his condition was complicated with severe gastric variceal bleeding due to splenic venous thrombosis, which was successfully treated via splenectomy and short gastric vein ligation. On the basis of our findings, we concluded that the splenic venous thrombosis-induced gastric variceal bleeding was a rare but life-threatening adverse effect of the therapeutic SI administered for primary splenic DLBCL. Surgical intervention involving splenectomy and short gastric vein ligation is mandatory and should be performed as soon as possible for such patients.

## Background

Diffuse large B-cell lymphoma (DLBCL) is the most common subtype of non-Hodgkin’s lymphoma, accounting for approximately 30% of cases [1]. A combination of rituximab (R) and the cyclophosphamide, adriamycin, vincristine, and prednisolone (CHOP) regimen has become the standard treatment for DLBCL because it greatly improves the outcome of patients with this disease, especially of young patients in the low-risk group [2]. Primary splenic DLBCL is a rare clinical condition, for which the treatment option generally involves six to eight cycles of chemotherapy with the R-CHOP regimen [3]. Furthermore, Iriyama *et al*. [4] reported the case of a primary splenic DLBCL patient who was successfully treated by splenectomy followed by three cycles of chemotherapy with the R-CHOP regimen, suggesting that surgery followed by abbreviated cycles of chemotherapy could be an effective alternative treatment for this disease. For patients who are refractory to chemotherapy or are inoperable, therapeutic splenic irradiation (SI) might be a reasonable and possibly the only treatment option with curative intention. However, the efficacy and safety of SI in treating primary splenic DLBCL remain uncertain and need further investigation. Herein, we present the case of a primary splenic DLBCL patient who was refractory to multiple chemotherapy regimens. Complete remission was achieved using therapeutic SI. However, his condition was complicated with severe gastric variceal bleeding due to splenic venous thrombosis (SVT), which was successfully treated via splenectomy and short gastric vein ligation.

## Case presentation

**Figure 1:**
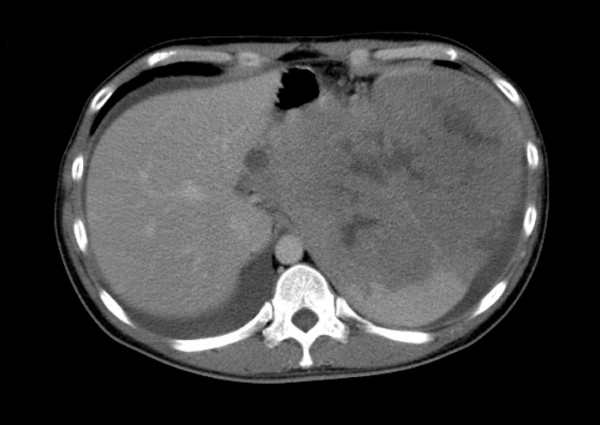
Computed tomography scan of the abdomen showed a large mass over the spleen and adjacent lymph nodes.

A 32-year-old man who was a hepatitis B carrier with Child-Pugh A liver cirrhosis presented with abdominal fullness of three-month duration. Abdominal computed tomography showed the presence of a large mass over his spleen and adjacent lymph nodes (Figure 1). Laboratory examinations for complete blood count yielded normal results (white blood cell count: 6100/mm^3^, normal range: 4000 to 8000/mm^3^; hemoglobin level: 12.7 g/dL, normal range: 12 to 16 g/dL; platelet count: 319000/mm^3^, normal range: 140000 to 400000/mm^3^; neutrophil: 65.3%; lymphocyte: 25.2%). The patient’s liver and renal functions were within the normal ranges. However, the level of lactate dehydrogenase was elevated (401 U/L, normal range: 140 to 200 U/L). Examinations for detecting coagulopathy yielded normal prothrombin time (10.8 s, normal range: 9.5 to 11.7 s), activated partial thromboplastin time (29.6 s, normal range: 24.3 to 32.7 s), protein C level (95.3%, normal range: 70 to 140%), protein S level (134%, normal range: 67 to 132%), and antithrombin III level (111%, normal range: 80 to 120%). Exploratory laparotomy and biopsy confirmed the diagnosis of DLBCL, which was immunohistochemically positive for CD20 and bcl6 but negative for CD3, CD30 and bcl2. The Ki-67 index was 80%. The tumor was staged as stage IIX because of the absence of bone marrow and chest involvement. The age-adjusted International Prognostic Index score was 2 [5].

**Figure 2:**
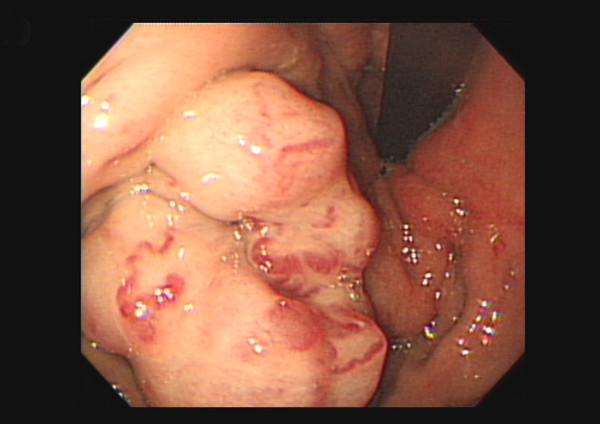
Upper gastrointestinal endoscopy image showed large gastric varices with a red color sign.

**Figure 3:**
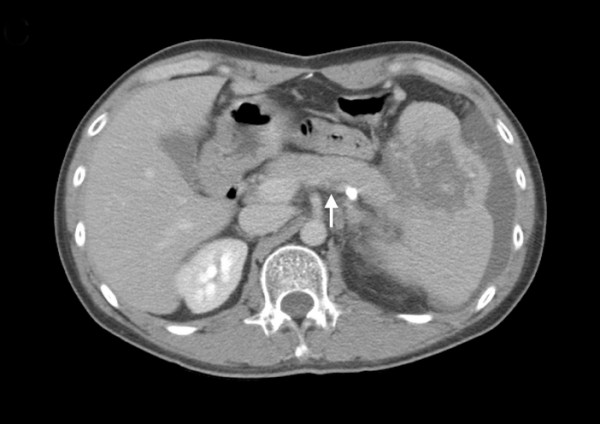
Abdominal computed tomography scan showed a filling defect in the splenic vein (arrow), indicating thrombosis formation.

The patient underwent six cycles of chemotherapy with the R-CHOP regimen; however, positron emission tomography (PET) and computed tomography detected a residual tumor over the spleen after therapy completion. The patient underwent autologous hematopoietic stem cell harvest after high-dose cyclophosphamide mobilization (2000 mg/(m^-2^·d^-1^) for 2 d); however, this procedure was not successful. Salvage chemotherapy involving two cycles of etoposide, cisplatin, methylprednisolone, and cytarabine (ESHAP) regimen was administered, but a residual tumor was still detected over his spleen via PET and computed tomography one month after the last ESHAP treatment. Subsequently, therapeutic SI comprising 2400 cGy in 15 fractions was administered. One month later, the patient developed upper gastrointestinal bleeding. Upper gastrointestinal endoscopy showed large gastric varices (GVs) with a red color sign (Figure 2) but the ultrasonography could not detect the splenic vein, which indicated the presence of SVT. Abdominal computed tomography further confirmed this diagnosis (Figure 3). Sclerotherapy by locally injecting N-butyl-2-cyanoacrylate could not stop the gastric variceal bleeding; therefore, we performed splenectomy and short gastric vein ligation. Pathological examination of the dissected spleen showed extensive necrosis, an organizing thrombus in the splenic vein, and no viable tumor cells. After surgical intervention, the GVs regressed completely, and the patient has been in complete remission for more than one year.

## Conclusions

Although the R-CHOP regimen has become the standard front-line treatment for DLBCL [6], the treatment for patients who are refractory to the R-CHOP regimen remains unclear. Salvage chemotherapy, autologous hematopoietic stem cell transplantation, or radiotherapy can be used as a therapeutic option for these patients [7]. Because our patient was refractory to salvage chemotherapy with the ESHAP regimen and autologous stem cell harvest was not successful, therapeutic SI was a reasonable and probably the only curative treatment for this patient. However, studies on the efficacy and adverse effects of therapeutic SI for primary splenic DLBCL are still required.

SI is a rarely used therapy. It is generally used for providing symptomatic relief to patients with chronic lymphoid leukemia or myeloproliferative neoplasm-related massive splenomegaly [8]. A palliative SI dose mostly ranges between 400 cGy and 1000 cGy with only slight toxicity [9]. Nevertheless, the optimal dose for therapeutic SI for primary splenic DLBCL is still not established clinically. Because doses of 3000 to 4000 cGy are usually required for treating DLBCL, our patient received therapeutic SI comprising a dose of 2400 cGy, which was much higher than that administered in a palliative setting. This treatment enabled the patient to achieve complete remission, suggesting that therapeutic SI comprising a dose of 2400 cGy could be effective for patients with chemorefractory splenic DLBCL.

Although our patient achieved complete remission via therapeutic SI, he suffered from a complication of SVT-associated gastric variceal bleeding. SI is believed to induce a broad range of both local and systemic effects, which are not yet completely understood [9]. Most of the reported adverse effects caused by SI are usually well-tolerated, mainly limited to myelosuppression, and are observed in palliative settings. However, the findings of a study conducted by Elliott *et al*. [10] showed that, after a single course of SI, prolonged, life-threatening pancytopenia occurred in 26% (6/23) of myelofibrosis patients. Moreover, three of the six patients in that cohort died of lethal sepsis or hemorrhage. Whether more severe myelosuppression occurs in patients who undergo therapeutic SI is not clear; however, it did not occur in our patient. In contrast, our patient’s condition was complicated with SVT-associated gastric variceal bleeding, which is life-threatening but rarely reported. The actual pathophysiology of SVT formation in patients who undergo therapeutic SI is not yet completely understood. Hypercoagulopathy disorders such as protein C, protein S, and antithrombin III deficiencies were absent in our patient, while treatment with chemotherapeutic agents [11], endoscopic sclerotherapy [12], malignant lymphoma [13], and post-hepatitis B liver cirrhosis were the factors applicable to our patient. The aforementioned conditions and factors can be risk factors for SVT formation in patients receiving SI.

In terms of treatment options for SVT-associated gastric variceal bleeding, balloon-occluded retrograde transvenous obliteration [14] and splenectomy with short gastric vein ligation could be both effective. Unfortunately, balloon-occluded retrograde transvenous obliteration was not available in our institution. In addition, our findings suggest that splenectomy with short gastric vein ligation should be mandatory when SVT-associated gastric variceal bleeding occurs after therapeutic SI.

In summary, therapeutic SI could be an effective treatment for chemorefractory primary splenic DLBCL. However, SVT-associated gastric variceal bleeding could be a probable but life-threatening adverse effect of this treatment. Surgical intervention involving splenectomy and short gastric vein ligation should be performed as soon as possible for such patients.

## Consent

Written informed consent was obtained from the patient for publication of this case report and any accompanying images. A copy of the written consent is available for review by the Editor-in-Chief of this journal.

## Abbreviations

CHOP, Cyclophosphamide, adriamycin, vincristine, and prednisolone; DLBCL, Diffuse large B-cell lymphoma; ESHAP, Etoposide, cisplatin, methylprednisolone, and cytarabine; GV, Gastric varix; PET, Positron emission tomography; R, Rituximab; SI, Splenic irradiation; SVT, Splenic venous thrombosis.

## Competing interests

The authors declare that they have no competing interests.

## Authors’ contributions

YCL participated in drafting the manuscript and in treating the patients. HCC participated in reading the images. WLH participated in treating the patients. SBC participated in treating the patients. RCW participated in pathology review. CJT (corresponding author) participated in drafting the manuscript and in treating the patients. All authors read and approved the final manuscript.
